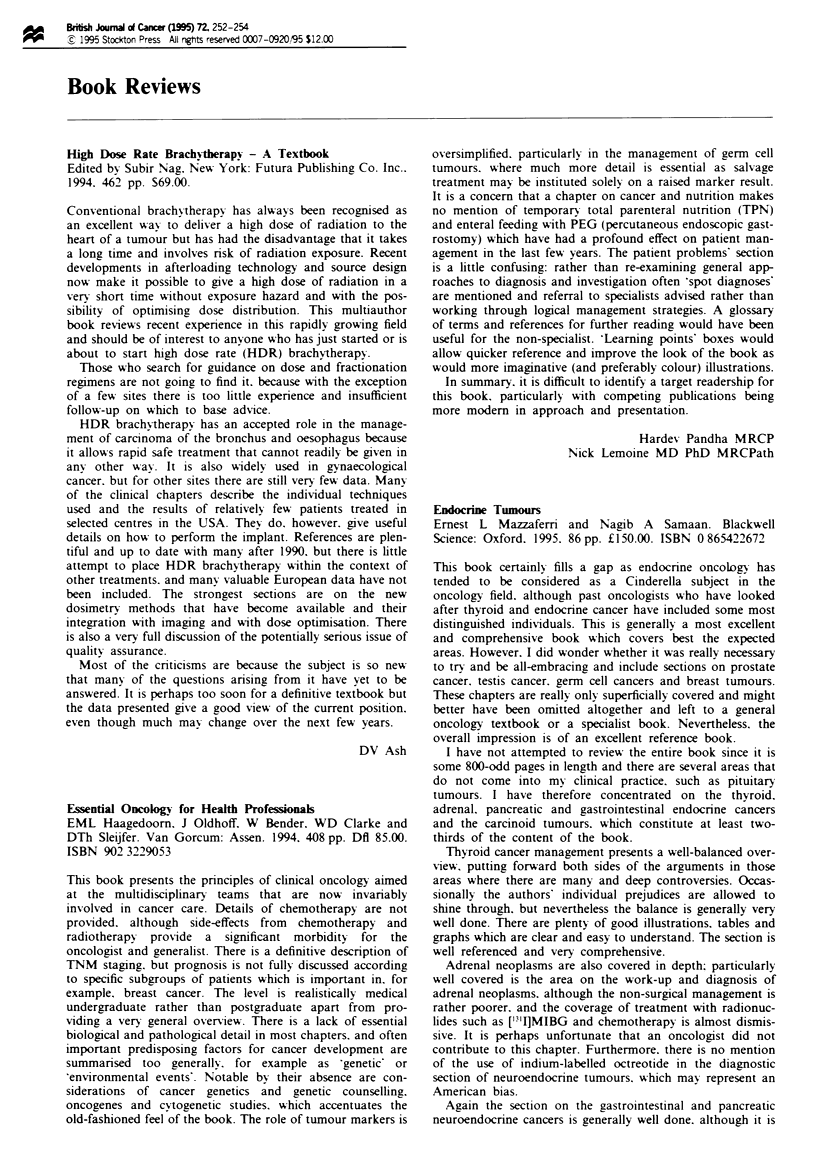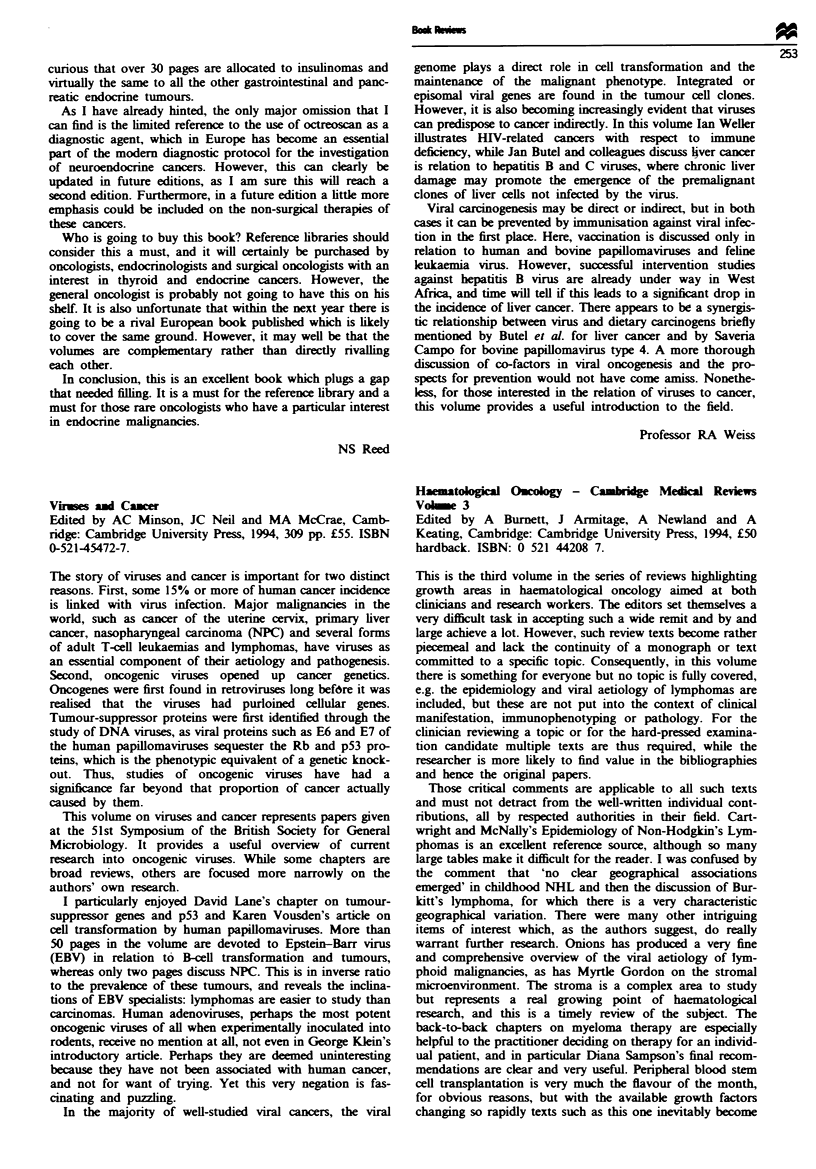# Endocrine Tumours

**Published:** 1995-07

**Authors:** NS Reed


**Endocrine Tumours**
Ernest L Mazzaferri and Nagib A Samaan. Blackwell Science: Oxford. 1995. 86 pp. £150.00. ISBN 0 865422672

This book certainly fills a gap as endocrine oncology has tended to be considered as a Cinderella subject in the oncology field, although past oncologists who have looked after thyroid and endocrine cancer have included some most distinguished individuals. This is generally a most excellent and comprehensive book which covers best the expected areas. However, I did wonder whether it was really necessary to try and be all-embracing and include sections on prostate cancer, testis cancer, germ cell cancers and breast tumours. These chapters are really only superficially covered and might better have been omitted altogether and left to a general oncology textbook or a specialist book. Nevertheless, the overall impression is of an excellent reference book.

I have not attempted to review the entire book since it is some 800-odd pages in length and there are several areas that do not come into my clinical practice, such as pituitary tumours. I have therefore concentrated on the thyroid, adrenal, pancreatic and gastrointestinal endocrine cancers and the carcinoid tumours, which constitute at least two-thirds of the content of the book.

Thyroid cancer management presents a well-balanced overview, putting forward both sides of the arguments in those areas where there are many and deep controversies. Occasionally the authors' individual prejudices are allowed to shine through, but nevertheless the balance is generally very well done. There are plenty of good illustrations, tables and graphs which are clear and easy to understand. The section is well referenced and very comprehensive.

Adrenal neoplasms are also covered in depth; particularly well covered is the area on the work-up and diagnosis of adrenal neoplasms, although the non-surgical management is rather poorer, and the coverage of treatment with radionuclides such as [$^{131}$I]MIBG and chemotherapy is almost dismissive. It is perhaps unfortunate that an oncologist did not contribute to this chapter. Furthermore, there is no mention of the use of indium-labelled octreotide in the diagnostic section of neuroendocrine tumours, which may represent an American bias.

Again the section on the gastrointestinal and pancreatic neuroendocrine cancers is generally well done, although it is curious that over 30 pages are allocated to insulinomas and virtually the same to all the other gastrointestinal and pancreatic endocrine tumours.

As I have already hinted, the only major omission that I can find is the limited reference to the use of octreoscan as a diagnostic agent, which in Europe has become an essential part of the modern diagnostic protocol for the investigation of neuroendocrine cancers. However, this can clearly be updated in future editions, as I am sure this will reach a second edition. Furthermore, in a future edition a little more emphasis could be included on the non-surgical therapies of these cancers.

Who is going to buy this book? Reference libraries should consider this a must, and it will certainly be purchased by oncologists, endocrinologists and surgical oncologists with an interest in thyroid and endocrine cancers. However, the general oncologist is probably not going to have this on his shelf. It is also unfortunate that within the next year there is going to be a rival European book published which is likely to cover the same ground. However, it may well be that the volumes are complementary rather than directly rivalling each other.

In conclusion, this is an excellent book which plugs a gap that needed filling. It is a must for the reference library and a must for those rare oncologists who have a particular interest in endocrine malignancies.

NS Reed